# A comparison of neuromuscular blockade and reversal using cisatricurium and neostigmine with rocuronium and sugamadex on the quality of recovery from general anaesthesia for percutaneous closure of left atria appendage

**DOI:** 10.1186/s13019-022-01936-1

**Published:** 2022-08-26

**Authors:** Qiongzhen Li, Haixia Yao, Jingxiang Wu, Meiying Xu, Hong Xie, Dongjin Wu

**Affiliations:** 1 Department of Anesthesia, The Second Affiliated Hospital of Soochow University, No. 1055, Sanxiang Road, Suzhou, 215004 China; 2 Department of Anesthesiology of Shanghai Chest Hospital, Shanghai Jiaotong University, Shanghai, 200030 China

**Keywords:** Left atrial appendage closure, Rocuronium, Sugammadex, Cisatracurium

## Abstract

**Background:**

There is a growing interest in minimally invasive left atrial appendage closure therapies. However, for successful catheter surgery, it is necessary to achieve high-quality postoperative recovery. The aim of the study is to comparison of neuromuscular blockade and reversal using cisatricurium and neostigmine with rocuronium and sugamadex on the quality of recovery from general anaesthesia for percutaneous closure of left atria appendage.

**Methods:**

Eighty-four patients who received percutaneous LAAC were randomly placed into two groups, general anesthesia and endotracheal intubation with either propofol-remifentanil-cisatracurium-neostigmine (group C) or propofol-remifentanil-rocuronium-sugammadex (group S). The QoR-40 questionnaire was used to assess recovery quality 6 h after surgery, and the time of spontaneous respiration, the time of consciousness recovery, the time of extubation, the duration in the postanaesthesia care unit (PACU), and the adverse events after awakening were collected.

**Results:**

Compared with the group C, the group S demonstrated significantly higher individual QoR-40 dimension scores, a significantly shorter recovery time for spontaneous respiration and consciousness, time of extubation, and duration in the PACU, and a lower incidence of transient hypoxemia, agitation, nausea and vomiting and urinary retention. There was a non-significant trend for the length of stay in the hospital in both groups.

**Conclusions:**

General anesthesia and endotracheal intubation with propofol-remifentanil-rocuronium-sugammadex provided better quality of recovery, shorter anaesthesia duration, and lower incidence of hypoxemia and agitation. Neuromuscular blockade and reversal using rocuronium and sugamadex is better than with cisatricurium and neostigmine on the quality of recovery from general anaesthesia for percutaneous closure of left atria appendage.

*Trial registration*: chictr.org, ChiCTR2000031857. Registered on April 12, 2020.

## Background

As one of the most common abnormal cardiac arythmia, atrial fibrillation (AF) affects approximately 5.5 million people worldwide, including 10% of individuals at least 75 years old [1, 2]. Currently, patients with a high stroke risk and contraindications to long-term oral anticoagulants [3, 4] are thought to have percutaneous left atrial appendage closure (LAAC). Because transoesophageal echocardiography (TEE) can be used as a significant imaging technique to guide the implantation of LAAC devices, general anaesthesia (GA) is very common in such procedures [5]. Recently, techniques guaranteeing quick and safe recovery from anaesthesia have become increasingly required.

As an integration of different perioperative patient care approaches, enhanced recovery after surgery (ERAS) combines evidence-based interventions that decrease surgical stress, maintain postoperative physiological function and promote the recovery of patients who have undergone major surgery [6]. ERAS protocols are mainly used to improve the early recovery of patients, which leads to shorter hospital stays without adversely influencing morbidity. Nevertheless, it is difficult to assess the recovery quality of patients with conventional recovery indicators, including time of awakening, duration of stay, or adverse events. The difference in the quality of recovery as a function of the type of anaesthesia or the use of adjuvant agents [7] is assessed by widely using the Quality of Recovery- 40 (QoR-40) questionnaire instead of these traditional approaches. Hence, to successfully perform percutaneous left atrial appendage closure LAAC surgery under general anaesthesia and endotracheal intubation, an anaesthetic approach with high-quality recovery should be selected.

With quick onset at higher doses, rocuronium has a prolonged duration of action. As a selective relaxant-binding agent, sugammadex allows the quick reversal of rocuronium-induced neuromuscular blockade [8]. Sugammadex, a modified cyclodextrin, acts as a selective relaxant-binding agent. Through generation of a tight complex with unbound steroidal NMBA molecules, sugammadex realizes a quick reversal of muscle relaxation, which stops their action at the neuromuscular junctions [9, 10]. Operating conditions may be improved by deep neuromuscular blockade during some surgical operations. Therefore, deep neuromuscular blockade can be reversed by sugammadex without waiting for spontaneous recovery [11].

General anaesthesia and endotracheal intubation with propofol-remifentanil rocuronium-sugammadex would improve the quality of recovery. The time of spontaneous respiration, the time of consciousness recovery, the time of extubation, the duration of stay in the post anaesthesia care unit (PACU), and the adverse events after awakening were determined. In addition, general anaesthesia is used have been provided comfortable for patients to tolerate the probe and its manipulations for a prolonged period. An optimal anaesthetic method was proposed for percutaneous left atrial appendage closure.

## Material and methods

### Research design and patient group

#### Patients and data collection

Eighty-four patients (60–80 years old) were enrolled in a randomized and prospective double-blind study from April 12, 2020 to June 12, 2020. The Institutional Review Board of Shanghai Jiaotong University approved the research protocol.

Shanghai Chest Hospital (KS1864) was chosen as the location for percutaneous left atrial appendage closures that require general anaesthesia. This study was registered at chictr.org (ChiCTR2000031857). Informed consent was signed by every patient participating in this research.

### Inclusion and exclusion standards

The participants were scheduled for percutaneous left atrial appendage closure surgery and received general anaesthesia and endotracheal intubation with either cisatracurium-neostigmine or rocuronium-sugammadex. The exclusion criteria included the following conditions: refusal to participate, use of psychiatric medications and alcohol abuse, a history of liver and kidney disease, contraindications to neostigmine and/or atropine or cognitive impairment.

### Blinding

The research team and the patients were not informed as to whether the patients were assigned to the propofol-remifentanil-cisatracurium-neostigmine (group C) and propofol-remifentanil-rocuronium-sugammadex (group S) until the end of the study. Only the dispensing nurse and clinical trial statistician know the group allocation.The blinding could be disrupted in case of emergency if the patients' health or safety were at risk.

### Preoperative preparations and anaesthesia protocol

The participants did not receive any premedication. After arriving in the operating room, the participants were monitored by pulse oximetry, electrocardiography (ECG), non-invasive blood pressure (NIBP), bispectral index and temperature. After inserting a catheter into a peripheral vein, the patients were injected with crystalloids of 6 ml/kg. The radial artery was cannulated to monitor invasive blood pressure after the administration of lidocaine local anaesthesia. The participants were randomly classified into groups C and S. A target-controlled infusion (TCI) of 2% propofol was used to induce anaesthesia with an effect-site concentration (Ce) of 4 μg/ml, together with 4 ng/ml remifentanil and 0.15 mg/kg cisatracurium in group C or with 0.9 mg/kg rocuronium in group S. Mask ventilation was initiated with 100% oxygen. A single-lumen tracheal catheter was used by experienced anaesthesiologists involved in the study to intubate patients with a tidal volume of 7 ml/kg, respiratory rate of 12 bpm, and I/E ratio of 1:2. Fifty percent oxygen with air was used to maintain PetCO_2_ was maintained at 35–45 mmHg. Then, the TEE probe was inserted. Anaesthesia was maintained with 2% propofol Ce at 1–2 μg/ml, together with 0.5–1.0 ng/ml remifentanil, 0.05 mg/kg cisatracurium as required in group C and 0.3 mg/kg rocuronium as required in group S every 30 min. The BIS was maintained between 40 and 50; if diastolic pressure fell below 90 mmHg, a phenylephrine intermittent bolus was administered; if the diastolic blood pressure rose above 140 mmHg, an intermittent bolus of nicardipine was administered. The nasopharyngeal temperature was maintained at ≥ 36 °C. After confirming that the left atrial appendage occluder was successfully placed, the puncture guide wire and the TEE probe were removed. All intravenous infusion anaesthetics were stopped using sugammadex 2–4 mg/kg as the sole reversal agent in group S. When the first spontaneous breath was taken by the patient, 0.02 mg/kg neostigmine and 10 μg/kg atropine were administered as reversal agents in group C. When the tracheal tube was removed, the patients were discharged to the PACU.

### Measurements

The primary outcome measures were the functional recovery quality 6 h after the operation. The secondary outcome measures were the time of spontaneous respiration, the time of consciousness recovery, the time of extubation, the duration of stay at the postanaesthesia care unit (PACU), and the adverse events after awakening, hypoxaemia (SpO2 < 90%), urinary retention (Unable to urinate autonomously), restlessness (cannot properly relax).

The QoR-40 questionnaire was used to assess the quality of functional recovery through interviews, including emotional state (9 items), physical comfort (12 items), physical independence (5 items), psychological support (7 items), and pain (7 items). A 5-point score was used to assess each item. The score of the QoR-40 questionnaire ranges from 40 (extremely poor) to 200 (excellent) [12].

### Statistical analysis

Based on past experimental results showing that patients who have received sugammadex recovered 3.4 times faster than patients who have received neostigmine, the sample size was calculated [11]. To achieve a study power of 90% (α = 0.05, β = 0.1), it was determined that the required sample size per group was 35. For a dropout rate of 20%, the final sample size was calculated at 42 patients in each group. The mean ± standard deviation is used to express quantitative variables. Categorical data are reported as frequencies and percentages. SPSS version 22 (SPSS, Chicago, IL) was used to perform statistical analyses. The χ^2^ test or Fisher's exact test was used to analyse all categorical variables. The Mann–Whitney U test or t-test was applied to test continuous variables depending on the distribution of the data. A *P* value < 0.05 was considered significant.

## Results

**Fig. 1:**
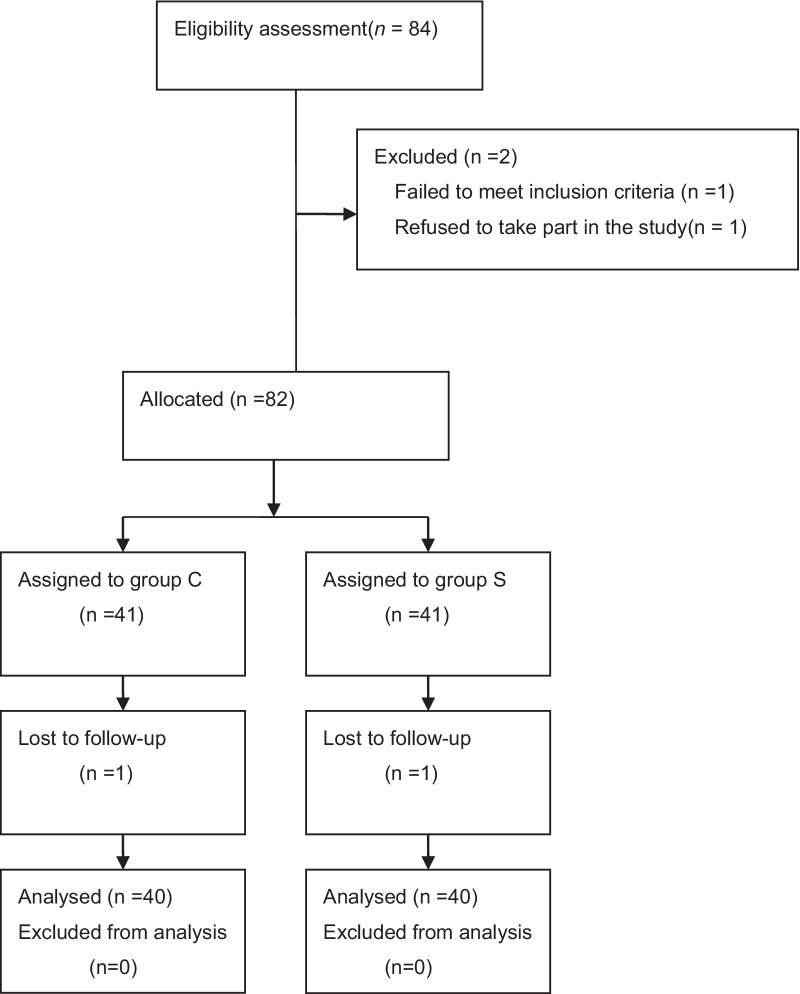
CONSORT flow diagram

This study enrolled 84 patients in total but excluded four patients. Therefore, the data from 80 patients were analysed (Fig. 1). The baseline features of the patients in the two groups were comparable (Table 1). The two groups were not significantly different in terms of age, BMI, gender, hypertension, diabetes, coronary heart disease, postoperative PCI, cerebral infarction, postoperative cardiovascular disease, or EF < 50% (*P* > 0.05, Table 1).

**Table 1:** Patient characteristics

Variable	Group C (n = 40)	Group S (n = 40)
Age (years)	75 ± 5	74 ± 4
BMI (kg/m^2^)	25 ± 3	25 ± 3
Gender (M/F)	24/16	28/12
Hypertension (%)	62.5	75
Diabetes (%)	37.5	22.5
Coronary heart disease (%)	37.5	35.0
Postoperative PCI (%)	22.5	25.0
Cerebral infarction (%)	37.5	40.0
Postoperative cardiovascular disease	35.0	25.0
EF < 50%	10.0	10.0

Data are expressed as the means ± SD or number (%)

The results for the intraoperative data are reported in Table 2. Although the length of stay in the hospital was similar between the groups (all *P* > 0.05), the time of the first spontaneous breath, eye opening, and extubation and the duration in the PACU were significantly shorter in the group S than in the group C.

**Table 2:** Intraoperative data

Variable	Group C (n = 40)	Group S (n = 40)	*P*
Time of first spontaneous breath (min)	22.2 ± 7.1	1.9 ± 0.6*	< 0.05
Time of eye opening (min)	28.8 ± 12.9	2.7 ± 0.6*	< 0.05
Time of extubation (min)	34.6 ± 16.0	3.6 ± 1.0*	< 0.05
Duration in the PACU	65 ± 20	26 ± 5*	< 0.05
Duration in the hospital (d)	4.0 ± 2.9	3.4 ± 1.2	> 0.05

Data are described as the means ± SD or number (%)

*P < 0.05 versus group C

Compared with those in group C, hypoxemia, nausea and vomiting, urinary retention and restlessness were significantly less frequent after surgery in the group S (*P* < 0.05, Table 3).

**Table 3:** Adverse effects after recovery

Variable	Group C (n = 40)	Group S (n = 40)	*P*
Hypoxemia, n (%)	7 (17.5)	1 (2.5)*	< 0.05
Nausea and vomiting, n (%)	3 (7.5)	0*	< 0.05
Urinary retention	10 (25.0)	2 (5.0)*	< 0.05
Restlessness n (%)	6 (15.0)	0*	< 0.05

Data are described as numbers (%)

*P < 0.05 vs group C

QoR-40 scores for cisatracurium-neostigmine(group C) or rocuronium-sugammadex (group S) on the day of surgery are presented in Table 4. Compared with group C, group S had a significantly higher total score (180.0 vs 168.0; *P* < 0.05) and significantly higher scores in emotional status and psychological support. There were significant differences between two groups in the other dimensions as well.

**Table 4:** Dimensions of the Quality of Recovery 40 (QoR–40) Questionnaire after surgery

	Group C (n = 40)	Group S (n = 40)	*P* values
Emotional status	49 (47–55)	55 (50–56)*	< 0.05
Physical comfort	41 (35–42)	42 (39–44)	> 0.05
Psychological support	20 (16–24)	23 (21–25)*	< 0.05
Physical independence	31 (27–33)	32 (27–35)	> 0.05
Pain	25 (20–33)	26 (21–34)	> 0.05
Total score	168 (154–183)	180 (163–190)*	< 0.05

Data are expressed as numbers (%) or medians (IQRs)

IQR, interquartile range; QoR-40, Quality of Recovery 40-item questionnaire

*P < 0.05 vs group C

## Discussion

An important finding of this study was strengthened global recovery quality among patients. To the best of our knowledge, this is the first study comparing the roles of various muscle relaxant/reversal agent regimens among patients who had undergone percutaneous left atrial appendage closure. This randomized clinical trial study showed that patients who underwent percutaneous left atrial appendage closure who received general anaesthesia and endotracheal intubation with rocuronium-sugammadex achieved better quality of recovery, a shorter anaesthesia duration and a lower incidence of hypoxemia and agitation than those who received general anaesthesia with cisatracurium-neostigmine. The emotional status and psychological support subcomponents in the quality of recovery instrument was also different between the two groups.

Another finding of this study was that anaesthesiologists must consider techniques for both quick and high-quality recovery that can minimize both minor morbidities and the time to return to daily activities as more surgeries are made in the clinical environment, such as ambulatory operations with high discharge rates. By impairing pulmonary and upregulating airway function, incomplete recovery of neuromuscular function may cause adverse respiratory events in the postanaesthesia care unit (PACU) [13].

In the present study, group S had a shorter anaesthesia duration than group C. As the anaesthesia time was reduced, the duration in the operating room was also reduced, and rapid surgical turnover was facilitated, which is beneficial for percutaneous left atrial appendage closure, which has a short time in the PACU. Patients may be at high risk of stroke during the perioperative period; therefore, it is important to judge their consciousness as soon as possible after the operation, as this would be beneficial for early detection and diagnosis of stroke. Hence, the quality of postoperative recovery in patients undergoing percutaneous left atrial appendage closure should be improved by taking appropriate measures. The propofol, remifentanil we used does not affect the rapid recovery of patients; instead, muscle relaxants have become the main drugs used to affect the recovery from anaesthesia. Sugammadex is a new type of selective steroid muscle relaxant antagonist that can quickly and completely antagonize rocuronium. Studies have confirmed that sugammadex is stable, safe and effective for cardiovascular disease patients during surgery. For people with high residual risk of adverse effects from muscle relaxants (such as obesity, old age, heart disease patients, lung disease patients, mild to moderate liver and kidney function impairment), no special dose adjustment is required [8, 10]. Based on a TCI of propofol and remifentanil, we used rocuronium as a muscle relaxant. At the end of the operation, we used sugammadex to antagonize the residual effect of rocuronium. After 1.9 ± 0.6 min spontaneous breathing recovery time, 2.7 ± 0.6 min to consciousness recovery, and 3.6 ± 1.0 min to tracheal tube removal, the group S demonstrated a significantly shortened PACU residence time (26 ± 5) min compared with 65 ± 20 min in group C, *P* < 0.05). The incidence of transient hypoxemia during the recovery period in group S was 2.5%, which was much less than that in group C (17.5%). Furthermore, the incidence of restlessness was 15.0% in group C, while group S has no restlessness. Rapid and conscious extubation increases patient comfort and reduces restlessness, allowing anaesthesiologists to deal with multiple cerebral infarctions before surgery, especially for patients with sequelae (language impairment, physical disability).

As an effective objective measurement of recovery quality after anaesthesia and surgery, the QoR-40 is the only evaluation tool meeting the eight quality-of-recovery criteria of appropriateness, reliability, validity, responsiveness, precision, interpretability, acceptability, and precision [14, 15] and is the best instrument to evaluate the complicated and multidimensional processes of postoperative recovery among general surgery patients. Compared with group C, group S had significantly higher global QoR-40 scores. According to several studies employing valid and reliable instruments, physical well-being, such as the presence of moderate or no pain or the absence of nausea, and mental well-being, including a feeling of general well-being, comfortableness and control, are significant elements in the quality of recovery after surgery and anaesthesia among the five dimensions in the QoR-40 [16], A dose of 2 mg/kg sugammadex was sufficient to reverse moderate rocuronium-induced neuromuscular blockade [17–19]. Phenomena such as less nausea and vomiting were observed among the patients who received sugammadex in our study, which may have strengthened their transition to physical comfort with respect to group C. According to early studies, neostigmine has been applied for reversal-caused postoperative nausea and vomiting [20, 21]. The most significant differences between the group S and C were in emotional status and psychological support. Except for the influence on PONV, each anaesthetic method differentially affecting the modulation of the stress response, which explains why psychological support was rated higher in group S in our research. Poor recovery exerts an adverse influence on both patients and the medical team. From the perspective of patients, their satisfaction with the medical services they received is lowered by a delayed return to normal activity, significant postoperative discomfort and a prolonged stay in the recovery room. Second, emotional status involves breathing, sleeping, eating, resting, PONV and shivering. Through seeking techniques that can achieve quick and smooth awakenings from general anaesthesia, the use of remifentanil and an opioid that undergoes rapid metabolism in combination with propofol should be considered.

This operation only requires femoral vein puncture, so local infiltration anaesthesia is sufficient for analgesia. The key steps of the surgical operation (such as atrial septum puncture and the release of the left atrial appendage occluder) require the patient to be absolutely still (no choking, body movement, etc.), so a deep degree of sedation is required to meet the needs of surgical safety. TEE guidance is required for the evaluation of the size and anatomy of the LAA and determination of optimal positioning of the LAAC instrument, which reduces patients’ pain and discomfort during the operation. Although some studies have reported that deep sedation anaesthesia can be used, the potentially fatal risks of deep sedation, such as upper airway obstruction and respiratory depression, require doctors to be prepared for emergency tracheal intubation at any time [22, 23]. Therefore, in the specific environment of the catheter room, where the patient must be monitored from outside the radiation shield or the partition window, controlling the airway remains a safer choice for the anaesthesiologists.

The current research has several limitations. First, this research did not evaluate the QoR-40 preoperatively. Nevertheless, the demographic data among patients showed no differences. Because only patients aged 60 to 80 were included, the history of the chronic use of antipsychotic medications was excluded. Second, limited by the study duration and other reasons, the sample size was not sufficiently large. In addition, the person and their experience assessing outcomes might have baring on the findings and possible assessment bias. Finally, this study failed to assess the surgical conditions because the main outcome was postoperative patient recovery.

## Conclusions

General anaesthesia and endotracheal intubation with rocuronium-sugammadex provided better quality of recovery, shorter anaesthesia duration, and a lower incidence of hypoxemia and agitation than cisatracurium-neostigmine. The former combination could be an optimal anaesthetic method for percutaneous left atrial appendage closure.

## Data Availability

Data sharing is not applicable for this article. The corresponding author will provide the trial data on reasonable request.

## Abbreviations

ASA: American Society of Anesthesiologists
LAA: Left atrial appendage
LAAC: Percutaneous left atrial appendage closure
BIS: Bispectral index
TEE: Transoesophageal echocardiography
QoR-40: Quality of Recovery 40
AF: Atrial fibrillation
GA: General anaesthesia
BMI: Body mass index
PCI: Percutaneous coronary intervention
EF: Left ventricular ejection fraction
IQR: Interquartile range
HR: Heart rate
ECG: Electrocardiography
MBP: Mean blood pressure
NIBP: Non-invasive blood pressure
SpO_2_: Pulse oxygen saturation
TCI: Target-controlled infusion
Ce: Effect-site concentration
I:E: Inspiration/expiration
PACU: Post-anaesthetic care unit
SD: Standard deviation
ERAS: Enhanced recovery after surgery
